# Analysis of Spleen-Induced Fimbria Production in Recombinant Attenuated *Salmonella enterica* Serovar Typhimurium Vaccine Strains

**DOI:** 10.1128/mBio.01189-17

**Published:** 2017-08-22

**Authors:** Paweł Łaniewski, Chang-Ho Baek, Kenneth L. Roland, Roy Curtiss

**Affiliations:** a The Biodesign Institute, Arizona State University, Tempe, Arizona, USA; b School of Life Sciences, Arizona State University, Tempe, Arizona, USA; Washington University School of Medicine

**Keywords:** Agf, Saf, Stc, Sti, fimbriae, *in vivo* expression, recombinant attenuated *Salmonella* vaccine

## Abstract

*Salmonella enterica* serovar Typhimurium genome encodes 13 fimbrial operons. Most of the fimbriae encoded by these operons are not produced under laboratory conditions but are likely to be synthesized *in vivo*. We used an *in vivo* expression technology (IVET) strategy to identify four fimbrial operons, *agf*, *saf*, *sti*, and *stc* that are expressed in the spleen. When any three of these operons were deleted, the strain retained wild-type virulence. However, when all four operons were deleted, the resulting strain was completely attenuated, indicating that these four fimbriae play functionally redundant roles critical for virulence. In mice, oral doses of as low as 1 × 10^5^ CFU of the strain with four fimbrial operons deleted provided 100% protection against challenge with 1 × 10^9^ CFU of wild-type *S*. Typhimurium. We also examined the possible effect of these fimbriae on the ability of a *Salmonella* vaccine strain to deliver a guest antigen. We modified one of our established attenuated vaccine strains, χ9088, to delete three fimbrial operons while the fourth operon was constitutively expressed. Each derivative was modified to express the *Streptococcus pneumoniae* antigen PspA. Strains that constitutively expressed *saf* or *stc* elicited a strong Th1 response with significantly greater levels of anti-PspA serum IgG and greater protective efficacy than strains carrying *saf* or *stc* deletions. The isogenic strain in which all four operons were deleted generated the lowest anti-PspA levels and did not protect against challenge with virulent *S. pneumoniae*. Our results indicate that these fimbriae play important roles, as yet not understood, in *Salmonella* virulence and immunogenicity.

## IMPORTANCE

*Salmonella enterica* is the leading cause of bacterial food-borne infection in the United States. S. Typhimurium is capable of producing up to 13 distinct surface structures called fimbriae that presumably mediate its adherence to surfaces. The roles of most of these fimbriae in disease are unknown. Identifying fimbriae produced during infection will provide important insights into how these bacterial structures contribute to disease and potentially induce protective immunity to *Salmonella* infection. We identified four fimbriae that are produced during infection. Deletion of all four of these fimbriae results in a significant reduction in virulence. We explored ways in which the expression of these fimbriae may be exploited for use in recombinant *Salmonella* vaccine strains and found that production of Saf and Stc fimbriae are important for generating a strong immune response against a vectored antigen. This work provides new insight into the role of fimbriae in disease and their potential for improving the efficacy of *Salmonella*-based vaccines.

## INTRODUCTION

Bacterial pathogens produce adhesins, often associated with fimbrial structures on the cell surface, to facilitate their initial interactions with host tissues (1). The chromosome of *Salmonella enterica* serovar Typhimurium contains 13 fimbrial operons, *agf* (*csg*), *bcf*, *fim*, *lpf*, *pef*, *saf*, *stb*, *stc*, *std*, *stf*, *sth*, *sti*, and *stj* (2–4). While the functions of a few of these fimbriae, including type 1 fimbriae (Fim), have been characterized (1, 5), the functions of most fimbriae are unknown. This is due, in part, to the fact that only type 1 and Agf fimbriae are produced under laboratory growth conditions (6). Type 1 fimbriae are produced when cells are grown at 37°C, and Agf fimbriae are produced when cells are grown at 26°C (7). While it is possible that some of these other fimbriae may be required for life outside a host (8), it is likely that many play an as yet undiscovered role in host interactions.

The *agf* operon encodes thin aggregative fimbriae (9) in *Salmonella*, and these fimbriae were later found to be similar to the fibronectin-binding surface structure known as curli (10) originally described in *Escherichia coli* (11). Thin aggregative fimbriae (hereafter Agf fimbriae) and curli are not produced *in vitro* at 37°C (11). Production of Agf fimbriae is typically induced in laboratory settings by growing cells at 26°C. Pef fimbriae mediate adherence to the murine small intestine and are required for fluid accumulation in infant mice. Expression of *pef* genes is regulated by DNA methylation (12). Stf fimbriae share homology with MR/P fimbriae of *Proteus mirabilis* and *E. coli* Pap fimbriae (13), and expression of *stfA* is induced during infection of bovine ileal loops (14). Long polar fimbriae (Lpf) are important for colonization of Peyer’s patches in mice by mediating adherence to M cells (5). Lpf also plays a role in the early stages of biofilm formation on host epithelial cells (15) and is involved in intestinal persistence (16). Lpf synthesis is regulated by an on-off switch mechanism (phase variation) to avoid host immune responses (17).

Some *S. enterica* fimbriae have been shown to serve functions beyond those required for interactions at the intestinal mucosal surface. For example, the Agf fimbriae are required for biofilm formation in the gallbladder (18, 19). In addition, the Stg fimbriae of *S. enterica* serovar Typhi, required for adherence to epithelial cells, also serves to inhibit phagocytosis (20). In S. Typhimurium, most fimbriae are produced *in vivo*, since mice immunized with S. Typhimurium produce antibodies against fimbrial subunits AgfA, BcfA, FimA, LpfA, PefA, StbA, StcA, StdA, StfA, SthA, and StiA (6). Thus, it is likely that some of these uncharacterized fimbriae may be synthesized in extraintestinal tissues.

To investigate potential roles for *S*. Typhimurium fimbriae in the host, we utilized an *in vivo* expression technology (IVET) strategy (21). We identified four fimbrial operons that are actively expressed in the spleen, only one of which, *agf*, is synthesized during *in vitro* growth (at 26°C). We characterized the impact of deletion and constitutive expression of all four fimbriae on virulence and immunogenicity.

## RESULTS

### Identification of fimbrial operons expressed in the spleen by IVET.

**FIG 1:**
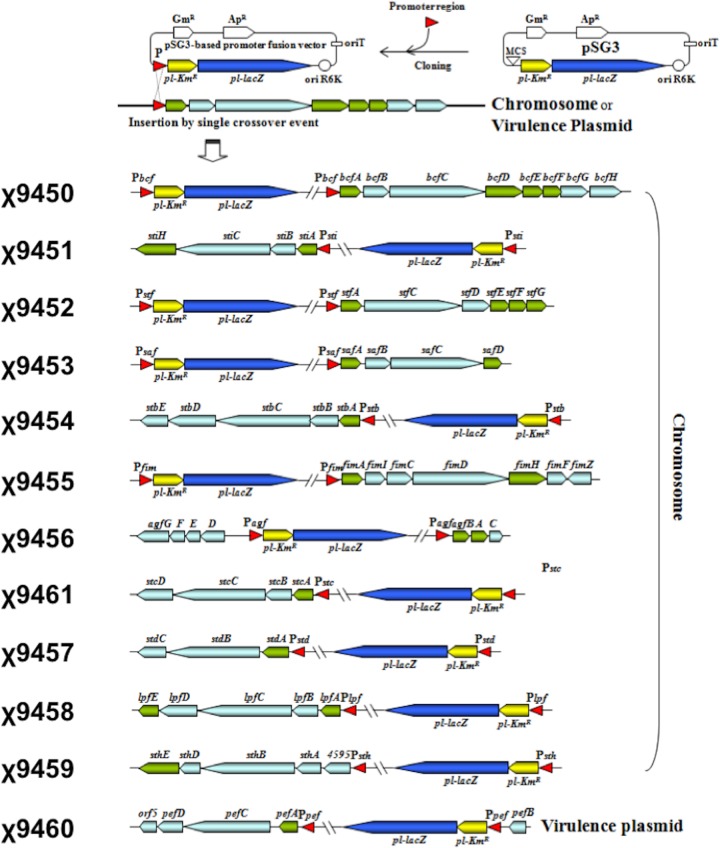
Construction of pSG3-based *aph lacZ* fusions in *S*. Typhimurium fimbrial operons. Promoters were isolated as PCR products ranging from 271 to 391 bp and cloned into plasmid pSG3 to construct chromosomal fusions of each fimbrial operon promoter to *aph*. The resulting promoter fusions are illustrated. Strains are resistant to kanamycin only when the corresponding fimbrial promoter is active.

We constructed 12 *S*. Typhimurium strains, each harboring chromosomal transcriptional fusions of fimbrial promoter regions with *aph lacZ* reporter genes (Fig. 1). The *stj* operon is incomplete due to the apparent absence of any identifiable fimbrial subunit genes, so it was not included in our study (2). However, it is likely that this operon encodes a nonfimbrial or fibrillar structure (4). A mixture of all 12 fusion strains were orally administered to BALB/c mice. After infection, mice were treated orally and intraperitoneally with three doses of kanamycin to select for *S*. Typhimurium clones expressing the *aph* reporter gene *in vivo*. The experiment was performed twice, and 96 clones were obtained from pooled spleen samples in each experiment. Clones were identified by PCR using specific primers (see Table S2 in the supplemental material). In both experiments, we recovered the same four *S*. Typhimurium strains, strains χ9451, χ9453, χ9456, and χ9461, which contain *aph lacZ* reporter genes fused to the promoter regions of *stiABCH*, *safABCD*, *agfBAC*, and *stcABCD* operons, respectively (Table 1). Each of these strains was sensitive to kanamycin when grown at 37°C on LB agar plates, indicating that these four fimbrial operons are expressed in the mouse host.

**TABLE 1:** Identification of S. Typhimurium fimbrial operons expressed in spleen using *in vivo* expression technology

Strain	Relevant reporter fusion for IVET	No. of PCR-positive clones (%) detected in spleen after kanamycin treatment (*n* = 96)	
		Expt 1	Expt 2
χ9450	P_*bcf*_::pl-*aph* pl-*lacZ*	0	0
χ9451	P_*sti*_::pl-*aph* pl-*lacZ*	42 (44)	10 (10)
χ9452	P_*stf*_::pl-*aph* pl-*lacZ*	0	0
χ9453	P_*saf*_::pl-*aph* pl-*lacZ*	19 (20)	59 (61)
χ9454	P_*stb*_::pl-*aph* pl-*lacZ*	0	0
χ9455	P_*fim*_::pl-*aph* pl-*lacZ*	0	0
χ9456	P_*agf*_::pl-*aph* pl-*lacZ*	26 (27)	7 (7)
χ9457	P_*std*_::pl-*aph* pl-*lacZ*	0	0
χ9458	P_*lpf*_::pl-*aph* pl-*lacZ*	0	0
χ9459	P_*sth*_::pl-*aph* pl-*lacZ*	0	0
χ9460	P_*pef*_::pl-*aph* pl-*lacZ*	0	0
χ9461	P_*stc*_::pl-*aph* pl-*lacZ*	5 (5)	17 (18)
Unidentified		4	3

### Virulence and immunogenicity of *S*. Typhimurium fimbrial mutants in BALB/c mice.

Because these fimbrial promoters are active in the spleen, we hypothesized that the fimbriae may be important for virulence. Therefore, we constructed strains harboring single and multiple deletions of the four fimbrial operons, Δ*stiABCH1225*, Δ*safABCD31*, Δ(*agfC-agfG*)-*999*, and Δ*stcABCD36*. BALB/c mice were orally administered graded doses of bacteria and monitored for 4 weeks. All single deletion mutants retained wild-type virulence (data not shown). Strains with any three of the four fimbrial operons deleted were also virulent (Table 2). In contrast, two independently constructed quadruple deletion mutants, χ11484 and χ11599, were fully attenuated, with no deaths or disease symptoms occurring at the highest dose tested (50% lethal dose [LD_50_] > ~1 × 10^9^  to 2 × 10^9^ CFU).

**TABLE 2:** Virulence of *S*. Typhimurium fimbrial mutants in BALB/c mice^a^

Strain	Relevant genotype	Oral LD_50_ (CFU)	
		Expt 1	Expt 2
χ3761	Wild-type	3.5 × 10^2^	NT^b^
χ11467	Δ(*agfC-agfG*)*-999* Δ*safABCD31* Δ*stcABCD36*	4.2 × 10^3^	5.1 × 10^2^
χ11483	Δ*safABCD31* Δ*stiABCH1225* Δ(*agfC-agfG*)*-999*	1.0 × 10^3^	4.1 × 10^2^
χ11505	Δ*safABCD31* Δ*stiABCH1225* Δ*stcABCD36*	5.6 × 10^2^	4.0 × 10^2^
χ11507	Δ(*agfC-agfG*)*-999* Δ*stiABCH1225* Δ*stcABCD36*	1.8 × 10^2^	1.2 × 10^3^
χ11484	Δ*safABCD31* Δ*stiABCH1225* Δ(*agfC-agfG*)*-999* Δ*stcABCD36*	>1.2 × 10^9^	>2.0 × 10^9^
χ11599	Δ*safABCD31* Δ*stiABCH1225* Δ*stcABCD36* Δ(*agfC-agfG*)*-999*	>1.6 × 10^9^	>1.3 × 10^9^

a BALB/c mice were orally administered graded doses of the indicated strains and monitored for 4 weeks.

b NT, not tested.

We evaluated the immunogenicity of one of the strains, χ11484, by determining its ability to confer protection against challenge with the virulent *S*. Typhimurium UK-1 strain χ3761. The mice used in the virulence assay (above), which received graded doses of strain χ11484 were challenged 4 weeks after immunization with *S*. Typhimurium χ3761 (Table 3). A control group was given sterile buffer. Protection was achieved at all doses, and all mice that were immunized with at least 1.4 × 10^5^ CFU of χ11484 survived challenge with the virulent *S*. Typhimurium strain (Table 3). Even mice inoculated with a single dose of only 8.4 × 10^2^ CFU were partially protected, indicating that this avirulent *S*. Typhimurium fimbrial quadruple mutant is highly immunogenic.

**TABLE 3:** Immunogenicity of *S*. Typhimurium fimbrial quadruple mutant in BALB/c mice^a^

Strain	Dose of χ11484 (CFU)	No. of mice alive after inoculation with χ11484/total no.	No. of mice alive after challenge with χ3761/ total no. (% survival)
χ11484	1.4 × 10^9^	6/6	6/6 (100)
	1.4 × 10^7^	6/6	6/6 (100)
	1.4 × 10^5^	6/6	6/6 (100)
	8.4 × 10^2^	6/6	4/6 (67)

None (control)			0/3 (0)

a BALB/c mice were immunized orally with the indicated dose of strain χ11484 (all mice survived) and challenged 4 weeks after immunization with ~1 × 10^9^ CFU of *S*. Typhimurium wild-type strain (χ3761).

### Colonization by *S*. Typhimurium fimbrial quadruple mutants.

**FIG 2:**
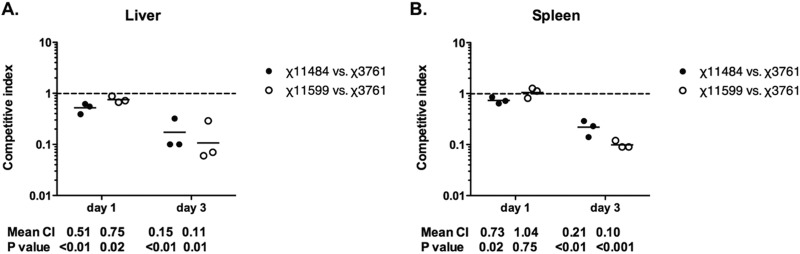
Effect of *saf sti stc agf* quadruple deletion on the colonization of mouse liver (A) and spleen (B) by *S*. Typhimurium. The competitive indexes were determined from mixed intraperitoneal infection with *S*. Typhimurium wild-type strain (χ3761) and one of two fimbrial quadruple mutants (χ11484 and χ11599). Each symbol represents the value for an organ from an individual mouse at the indicated day following the infection. The geometric means of the competitive indexes (mean CI) and the *P* values from a Student’s *t* test are given below the graphs.

To evaluate the impact of the quadruple deletion on colonization, mice were orally inoculated with either strain χ11484 or strain χ11599. Peyer’s patches, spleens, and livers were harvested 5 days later, and the bacteria in each tissue were enumerated. Both quadruple mutants colonized all tested organs as well as wild-type χ3761 strain did (data not shown). To look more closely at spleen and liver colonization, we performed a competition assay. We chose to inoculate by the intraperitoneal route to eliminate any differences between strains that might be due to passage through the gastrointestinal tract. Thus, mice were inoculated parenterally with a mixture of *S*. Typhimurium wild-type χ3761 and either χ11484 or χ11599. Each strain was marked with a stable low-copy-number chloramphenicol-resistant plasmid (pHSG576) or kanamycin-resistant plasmid (pWSK129). Groups of mice were euthanized on days 1 and 3 postinfection. Samples of the spleens and livers were plated for enumeration of *Salmonella*. The total numbers of *Salmonella* recovered from each organ were consistent from mouse to mouse, between 10^4^ and 10^6^ CFU per g of tissue (data not shown). The ratio of the two strains in each organ was determined and compared to the input ratio to determine the competitive index (CI). On day 1 postinfection, there were no differences in spleen colonization between wild-type and mutant strains (Fig. 2B), while strain χ11484, but not χ11599, was outcompeted by the wild-type strain in the liver (Fig. 2A) (*P* < 0.01). By day 3, the wild type had outcompeted both quadruple mutants in both the spleen and liver (*P* < 0.05), indicating an important role for *saf*, *sti*, *stc*, and *agf* in colonization of the spleen and liver in mice. In preliminary competition experiments comparing single deletion mutants and the wild type, no significant differences were observed between strains (data not shown), indicating that no single fimbria is responsible for this phenotype.

### Recombinant attenuated *S*. Typhimurium vaccine (RASV) strains producing fimbriae (Saf^+^, Sti^+^, Stc^+^, and Agf^+^) in a constitutive manner.

Our results showing that the *saf*, *sti*, *stc* and *agf* fimbrial operons are expressed *in vivo* led us to speculate as to whether these fimbriae could be exploited to enhance the immunogenicity and protective efficacy of *Salmonella* vaccine strains. For this work, we constructed derivatives of attenuated *S*. Typhimurium strain χ9088 [ΔP_fur33_::TT *araC* P_BAD_ *fur* Δ*pmi-2426* Δ(*gmd*-*fcl*)*-26* Δ*asdA33*] (22) in which three fimbrial operons were deleted and the fourth was expressed from the constitutive P_*murA*_ promoter (23). Consequently, the resulting strains, strains χ11595, χ11850, and χ11851, have a genetic background that includes attenuating mutations, deletions in three fimbrial operons, and one deletion-insertion mutation (Table 4). The *agf* genes are expressed from two divergent operons, *agfDEFG* and *agfBAC*, necessitating a different strategy. In this case, we introduced the previously described *agfD812* mutation (24) to drive constitutive expression of the *agf* operon. Strain χ12038 constitutively produced Agf fimbriae as indicated by the red, dry, and rough (rdar) colony morphology when grown on Congo red plates (7) at 37°C (data not shown). For a control, we also constructed strain χ11606, which harbors deletions of all four fimbrial operons (*agf*, *saf*, *sti*, and *stc*).

**TABLE 4:** Key bacterial strains and plasmids used in this study

Bacterial strain or plasmid	Relevant characteristic(s)	Reference or source
*E. coli* strains		
DH5α	F^−^ φ80*lacZ*ΔM15 Δ(*lacZYA*-*argF*)*U169 recA1 endA1 hsdR17*(r_K_^−^ m_K_^+^) *phoA supE44* λ^−^ *thi*-*1 gyrA96 relA1*; used for general cloning	51
BL21(DE3)	F^−^ *ompT hsdS*_B_(r_B_^−^ m_B_^−^) *gal dcm* (DE3); used for protein overproduction	Novagen
χ6212	F^−^ λ^−^ φ80 Δ(*lacZYA-argF*) *endA1 recA1 hsdR17 deoR thi-1 glnV44 gyrA96 relA1* Δ*asdA4*	52
χ7213	*thi-1 thr-1 leuB6 glnV44 fhuA21 lacY1 recA1* RP4-2-Tc::Mu(λ*pir*) Δ*asdA4* Δ(*zhf-2*::Tn*10*)	46

*S*. Typhimurium strains		
χ3761	Wild-type UK-1	45
χ9088	Δ*pmi-2426* Δ(*gmd*-*fcl*)-*26* ΔP_fur33_::TT *araC* P_BAD_ *fur* Δ*asdA33*	22
χ11467	Δ(*agfC-agfG*)*-999* Δ*safABCD31* Δ*stcABCD36*	χ11466
χ11483	Δ*safABCD31* Δ*stiABCH1225* Δ(*agfC-agfG*)*-999*	χ11468
χ11484	Δ*safABCD31* Δ*stiABCH1225* Δ(*agfC-agfG*)*-999* Δ*stcABCD36*	χ11483
χ11505	Δ*safABCD31* Δ*stiABCH1225* Δ*stcABCD36*	χ11468
χ11507	Δ(*agfC-agfG*)*-999* Δ*stiABCH1225* Δ*stcABCD36*	χ11506
χ11595	Δ*pmi-2426* Δ(*gmd-fcl*)-*26* ΔP_fur33_::TT *araC* P_BAD_ *fur* Δ*asdA33* Δ*safABCD31* Δ(*agfC-agfG*)*-999* Δ*stcABCD36* ΔP_*stiA52*_::P_*murA*_ *stiA52*	χ11594
χ11599	Δ*safABCD31* Δ*stiABCH1225* Δ*stcABCD36* Δ(*agfC-agfG*)*-999*	χ11505
χ11606	Δ*pmi-2426* Δ(*gmd-fcl*)-*26* ΔP_fur33_::TT *araC* P_BAD_ *fur* Δ*asdA33* Δ*safABCD31* Δ*stiABCH1225* Δ(*agfC-agfG*)*-999* Δ*stcABCD36*	χ11597
χ11850	Δ*pmi-2426* Δ(*gmd-fcl*)-*26* ΔP_fur33_::TT *araC* P_BAD_ *fur* Δ*asdA33* Δ*stiABCH1225* Δ(*agfC-agfG*)*-999* Δ*stcABCD36* ΔP_*safA55*_::P_*murA*_ *safA55*	χ11594
χ11851	Δ*pmi-2426* Δ(*gmd-fcl*)-*26* ΔP_fur33_::TT *araC* P_BAD_ *fur* Δ*asdA33* Δ*safABCD31* Δ*stiABCH1225* Δ(*agfC-agfG*)*-999* ΔP_*stcA53*_::P_*murA*_ *stcA53*	χ11597
χ12038	Δ*pmi-2426* Δ(*gmd-fcl*)-*26* ΔP_fur33_::TT *araC* P_BAD_ *fur* Δ*asdA33* Δ*safABCD31* Δ*stiABCH1225* Δ*stcABCD36 agfD812*	χ11562

*S. pneumoniae* strain WU2	Wild-type; virulent; encapsulated type 3	41

Plasmids		
pSG3	IVET vector; promoterless *aph lacZ mobRP4*; R6K ori; Ap^r^ Gm^r^	43
Plasmids used for production of recombinant proteins		
pET28b	Expression vector; T7 promoter *6xHis lacI*; f1 pBR *ori*; Km^r^	Novagen
pYA4085	pET30a derivative for overproduction of rPspA	49
pYA4088	pYA3493 derivative for production of rPspA (amino acids 3 to 285) fused to β-lactamase signal sequence	25

To study the ability of these strains to elicit protective immune responses against heterologous antigens in mice, we introduced plasmid pYA4088 (25), carrying the gene encoding the *Streptococcus pneumoniae* protein PspA, into each strain. This pneumococcal protein has been extensively studied by our group (26) and others (27, 28) and shown to elicit protective immunity against virulent *S. pneumoniae* challenge. For clarity, we will refer to these strains as χ11595(pYA4088) (Sti^+^), χ11850(pYA4088) (Saf^+^), χ11851(pYA4088) (Stc^+^), χ12038(pYA4088) (Agf^+^), and χ11606(pYA4088) (Δ4). All strains were grown to mid-log phase in LB with appropriate supplements. Western blot analysis with specific anti-recombinant PspA (anti-rPspA) antibodies showed that all strains produced similar amounts of PspA (Fig. S1).

10.1128/mBio.01189-17.1 FIG S1  Production of PspA antigen in S. Typhimurium RASV strains. Western blot showing whole-cell lysates obtained from mid-log-phase cultures, electrophoresed on a 12% SDS-polyacrylamide gel, transferred onto nitrocellulose, and probed with anti-rPspA serum. Download FIG S1, TIF file, 0.2 MB. Copyright © 2017 Łaniewski et al. 2017 Łaniewski et al. This content is distributed under the terms of the Creative Commons Attribution 4.0 International license.

### Antibody responses in mice immunized with RASV strains constitutively producing individual fimbriae (Saf^+^, Sti^+^, Stc^+^, and Agf^+^).

**FIG 3:**
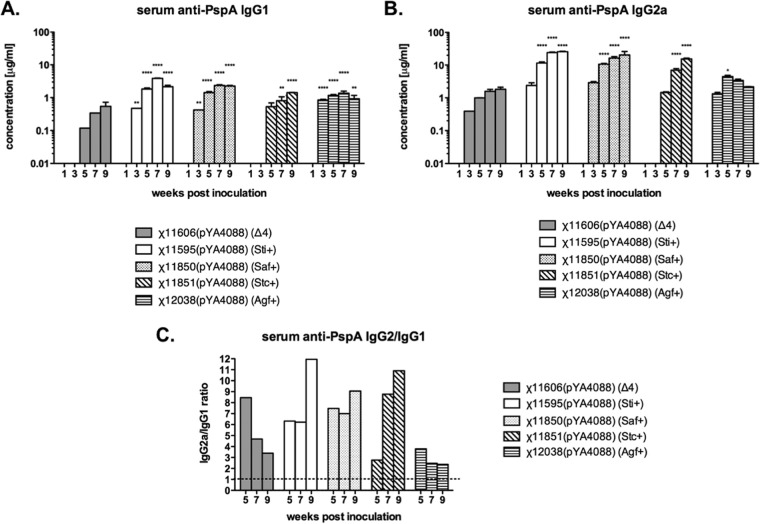
Serum IgG1 and IgG2a responses to PspA in mice immunized with RASV strains expressing fimbriae in a constitutive manner (Saf^+^, Sti^+^, Stc^+^, and Agf^+^) and producing PspA antigen. The kinetics of serum IgG1 and IgG2a responses to PspA in mice are shown. The data represent the concentrations of anti-PspA IgG1 (A) and IgG2a (B) in pooled serum samples from eight mice measured in duplicate. Error bars show the differences between the duplicates (standard deviations). All samples from immunized mice were significantly different from those from the control group given PBS (*P* < 0.05). Values that are significantly different from the values for the *S*. Typhimurium χ11606(pYA4088) group are indicated by asterisks as follows: *, *P* < 0.05; **, *P* < 0.01, ****, *P* < 0.0001. (C) Calculated IgG2a/IgG1 ratios based on the data shown in panels A and B.

BALB/c mice were orally primed and boosted 6 weeks later with identical doses of ~1 × 10^8^ CFU of each strain. A control group was given sterile buffer instead of vaccine. All mice immunized with RASVs expressing *pspA* produced anti-rPspA serum IgG1 (Fig. 3A) and IgG2a (Fig. 3B). No anti-rPspA IgG1 or IgG2a was detected in sera from control mice treated with phosphate-buffered saline (PBS). The anti-PspA serum IgG1 titers in all immunized mice were significantly higher than the titers in mice immunized with strain χ11606(pYA4088) (Δ4) by week 3 (Fig. 3A). The IgG2a subclass concentrations were also greater than in the χ11606(pYA4088) group in all cases except the group immunized with strain χ12038(pYA4088) (Agf^+^) (Fig. 3B). By week 9, IgG2a concentrations were 8- to 14-fold higher in mice immunized with strains χ11595(pYA4088) (Sti^+^), χ11850(pYA4088) (Saf^+^), and χ11851(pYA4088) (Stc^+^) than in mice immunized with strain χ11606(pYA4088) (Δ4) (*P* < 0.0001) (Fig. 3B). The IgG1 subclass concentrations for these three strains at week 9 were also elevated (2.6- to 4.2-fold) compared to those of χ11606(pYA4088) (Δ4) (*P* < 0.0001) (Fig. 3A). Comparing the anti-PspA IgG2a/IgG isotype ratios showed that immunization with each strain induced a mixed Th1/Th2 response, with a strong Th1 bias (Fig. 3C). At week 9, mice immunized with χ11595(pYA4088) (Sti^+^), χ11850(pYA4088) (Saf^+^), and χ11851(pYA4088) (Stc^+^) showed the highest IgG2a-to-IgG1 ratios, ranging from 9 to 12 (Fig. 3C). In contrast, mice immunized with χ11606(pYA4088) (Δ4) or χ12038(pYA4088) (Agf^+^) showed only two- to threefold differences in IgG2a-to-IgG1 titers. However, these differences were not statistically significant (*P* > 0.05).

### Protection of mice immunized with RASV strains constitutively producing individual fimbriae (Saf^+^, Sti^+^, Stc^+^, and Agf^+^) against *S. pneumoniae* challenge.

Four weeks after the boost, mice were injected intraperitoneally with ~40 times the LD_50_ of virulent *S. pneumoniae* strain WU2. Immunization with strain χ11850(pYA4088) (Saf^+^) and strain χ11851(pYA4088) (Stc^+^) provided the highest level of protection (52.6%) compared to nonimmunized control mice (*P* < 0.001) (Table 5). Strain χ11595(pYA4088) (Sti^+^) also provided significant protection against pneumococcal challenge (31.6%; *P* < 0.05). Vaccination with strain χ12038(pYA4088) (Agf^+^) resulted in 26.3% survival, but this result was not significantly different from that with the nonimmunized control group. In addition, mice vaccinated with strain χ11606(pYA4088) (Δ4) were not protected (10.5% survival). Two independent protection experiments were performed. All deaths occurred 4 to 6 days postinfection.

**TABLE 5:** Protective efficacy of RASV strains expressing fimbriae in a constitutive manner (Saf^+^, Sti^+^, Stc^+^, and Agf^+^) and producing PspA antigen^a^

Strain	Constitutively expressed fimbrial gene	No. of mice alive/total no. (% survival)		
		Expt 1	Expt 2	Combined^b^
χ11595(pYA4088)	*sti*	4/8 (50)^c^	2/11 (18.2)	6/19 (31.6)^c^
χ11850(pYA4088)	*saf*	5/8 (62.5)^d^	5/11 (45.5)	10/19 (52.6)^e^
χ11851(pYA4088)	*stc*	5/8 (62.5)^d^	5/11 (45.5)	10/19 (52.6)^d^
χ12038(pYA4088)	*agf*	3/8 (37.5)	2/11 (18.2)	5/19 (26.3)
χ11606(pYA4088) (Δ4)		1/8 (12.5)	1/11 (9.1)	2/19 (10.5)
χ9088(pYA4088)		NT^f^	3/11 (27.3)	3/11 (27.3)

None (PBS) (control)		0/8 (0)	0/11 (0)	0/18 (0)

a Seven-week-old BALB/c mice were immunized orally with ~1 × 10^8^ CFU of the indicated of *S*. Typhimurium vaccine strains and boosted with the same dose 6 weeks later. All mice were challenged by intraperitoneal inoculation 4 weeks after the booster dose with ~1 × 10^4^ CFU of virulent *S. pneumoniae* strain WU2. Deaths were recorded until 3 weeks postinfection.

b Combined percent survival from two independent experiments.

c Significantly different (*P* < 0.05) from value obtained for the control (PBS) group.

d Significantly different (*P* < 0.01) from value obtained for the control (PBS) group.

e Significantly different (*P* < 0.001) rom value obtained for the control (PBS) group.

f NT, not tested.

## DISCUSSION

Fimbrial genes are widely distributed among bacteria, but only a few fimbriae are produced under standard laboratory conditions. Most bacterial fimbriae serve to present adhesins that assist in the adherence of bacteria to biotic and abiotic surfaces (1) and are produced in response to the appropriate environmental cues. Of the 13 known fimbriae in *S*. Typhimurium, only two, type 1 fimbriae and curli (Agf) are readily produced when grown in the laboratory. However, in one study, cells were coaxed to produce Pef, Bcf, Stb, Stc, Std, and Sth fimbriae after static growth in CFA broth at 32°C and Agf, Pef, Lpf, Stc, Stf, and Sth fimbriae in LB at pH 5.1 at 37°C, although the levels were low, as fimbriae were detected on <7% of the cells by a highly sensitive flow cytometry method (14). In the same study, fimbrial expression was further enhanced to around 10% of cells after growth for 8 h in bovine ileal loops. Type 1 fimbriae were detected in >20% of the cells under all three growth conditions. Thus, it is likely that a majority of the known *S*. Typhimurium fimbrial operons are expressed inside a mammalian host.

In the current study, we demonstrated that *sti*, *saf*, *stc*, and *agf* fimbrial genes are actively expressed in the mouse spleen (Table 1). *In vivo* expression of these genes is consistent with a previous study in which CBA mice inoculated with *S*. Typhimurium developed antibodies against recombinant His-tagged StiA, StcA, and AgfA (6). The mice were not evaluated for antibody responses against Saf fimbrial components. In another study, mice were protected from challenge with *S*. Typhimurium after injection with a mixture of purified recombinant His-tagged SafB, a putative chaperone, and recombinant SafD, the Saf adhesin, both produced in *E. coli* (29, 30). In addition, transcription of *saf* fimbrial genes has been detected in blood samples from patients infected with S. Typhi (31) and *S*. Paratyphi A (32), supporting a role for these fimbriae in the human host.

SafA monomeric fimbriae were assembled *in vitro* in the presence of the chaperone protein SafB and crystallized (33). Subsequent crystallographic analysis showed that Saf fimbriae are composed of highly flexible fibers formed by globular subunits organized in a “beads on a string” arrangement (33). Characterization of the *safABCD* operon protein sequences suggest that SafA is the major structural protein, SafB is the periplasmic chaperone, and SafC is an outer membrane usher (29). The SafD protein is homologous to several other fimbrial adhesins and so is likely to be the Saf adhesin, believed to be present only at the tip of the fiber (29, 33). In addition, the major fimbrial protein, SafA, exhibits similarity to the λ phage-encoded Bor protein that has been implicated in serum resistance of λ-infected hosts (34). Thus, it is possible that the *saf* fimbriae play a role in serum resistance.

Our results with *agf* seem to run counter to a previous report. Using a bioluminescence imaging technique, White et al. showed that *agfB* was not expressed during infection (35). The authors concluded that Agf fimbriae are not produced *in vivo*. However, their observations were based on results obtained from a single time point, while in our study, the bacteria were under constant selective pressure for 3 days. Thus, it is possible that there is a temporal component to *agfB* expression. Our data suggesting the *in vivo* production of Agf is also supported by the study we cite above in which anti-AgfA antibodies were detected in *S*. Typhimurium-infected mice (6).

In a previous study, strains with either *agfAB* or *stcABCD* deleted exhibited wild-type levels of spleen colonization in genetically resistant CBA mice (16). Consistent with those results, we observed that strains in which any single fimbrial operon (*agf*, *saf*, *stc*, or *sti*) or combination of three operons was deleted had no effect on virulence, while deletion of all four fimbrial operons resulted in a complete loss of virulence when mutant strains were administered by the oral route to genetically sensitive BALB/c mice (Table 2). Our results suggest that these four fimbriae serve functionally redundant roles in mouse virulence. Interestingly, while a Δ*stcABCD* strain exhibits wild-type spleen colonization, it exhibits reduced fecal shedding, indicating a role for this fimbriae in long-term intestinal carriage (16).

Strain χ11484 with *sti*, *saf*, *stc*, and *agf* deleted was immunogenic, protecting mice from a high-dose challenge with wild-type *S*. Typhimurium after a single immunizing dose as low as 1.4 × 10^5^ CFU (Table 3). We expanded our analysis of the roles of these genes in immunogenicity by examining the effect of constitutive production of each fimbriae individually in a previously characterized vaccine strain background (χ9088) in which we had also deleted the other three fimbrial operons. These vaccine strains were used to deliver the heterologous antigen, PspA. Our results indicate that constitutive production of Sti, Saf, or Stc, but not Agf, significantly enhanced protective immunity (Table 5), although they each had different impacts on the immune system.

Th1-type dominant immune responses are frequently observed after immunization with attenuated *Salmonella* (36), and most of the fimbrial deletion strains elicited a Th1-biased response. However, mice immunized with strain χ12038(pYA4088) (Agf^+^) produced more of a mixed Th1/Th2 humoral response, indicating that overproduction of Agf fimbriae resulted in a reduced ability to stimulate Th1 helper cells to direct IgG class switching to IgG2a (37). IgG2a is the isotype with the greatest capacity to mediate complement deposition onto the surfaces of bacteria, and an increase in anti-PspA IgG2a has been correlated with increased C3 deposition on the *S. pneumoniae* cell surface (38).

The immune responses to PspA were examined by measuring the levels of IgG isotype subclasses. The anti-PspA IgG2a titers were higher than the IgG1 titers in all groups, indicating that all of the *Salmonella* vaccines induced a Th1-biased response against PspA (Fig. 3). Strain χ11850(pYA4088) (Saf^+^) elicited high levels of anti-PspA IgG with a strong Th1 bias (Fig. 3). Thus, the strong Th1 responses observed in mice vaccinated with strain χ11850(pYA4088) (Saf^+^) can explain why this strain was highly protective (Fig. 3 and Table 3). Strain χ11595(pYA4088) (Sti^+^) produced a strong Th1 response by week 9 (Fig. 3A). In contrast, the strains that provided the weakest protection, χ11606(pYA4088) (Δ4) and χ12038(pYA4088) (Agf^+^), were deficient in either strong Th1-biased antibody responses.

The strong protection observed for mice immunized with strain χ11851(pYA4088) (Stc^+^) does not fit as neatly into this interpretation, as this strain did not elicit a strong Th1 response at early time points (Fig. 3). However, by week 9, this strain elicited the greatest IgG2a/IgG1 ratio (Fig. 3C), which may have provided a humoral response that was adequate to control the *S. pneumoniae* challenge. This result, along with the results for strain χ12038(pYA4088) (Agf^+^), which stimulated a low IgG2a/IgG1 ratio, indicates that production of IgG2a is the most important parameter for protection against pneumococcal challenge in this model.

Deletion of all four fimbrial operons in strain χ11484 resulted in complete attenuation (Table 2), while preserving its ability to elicit a protective response against challenge with wild-type *S*. Typhimurium at immunizing doses as low as 8.4 × 10^2^ CFU (Table 3). In contrast, deletion of these same four fimbrial operons in the χ9088 background vectoring PspA compromised the ability of the strain to elicit protection against streptococcal challenge (Table 5). Since the Δ4 deletion is attenuating, combining these mutations with additional attenuating mutations could have resulted in overattenuation of the *Salmonella* vector strain, possibly due to a reduction in the ability of strain χ11606(pYA4088) to colonize the spleen or other lymphoid organs. While the basis of this overattenuation is not clear, it does indicate that one must carefully consider the background genotype before combining Δ4 with other attenuating mutations.

This study demonstrates that *in vivo*-induced fimbriae play a role in spleen colonization and may be used to augment the immunogenicity of orally administered, live attenuated *Salmonella* vaccines. This represents a novel strategy for modulating host immune responses to strengthen Th1-biased immune responses and enhance protective immunity.

## MATERIALS AND METHODS

### Bacterial strains, plasmids, and growth conditions.

Bacterial strains and plasmids used in this study are listed in Table 4 and Table S1 in the supplemental material. *Escherichia coli* and *Salmonella enterica* serovar Typhimurium strains were routinely cultured at 37°C in LB broth (39) or on LB agar. Cultures of *S*. Typhimurium strain χ9088 (22) and its derivatives were supplemented with 0.05% mannose (for Δ*pmi-2426*) and 0.2% arabinose (for ΔP_fur33_::TT *araC* P_BAD_ *fur*). Diaminopimelic acid (DAP) (50 µg/ml) was added to LB medium for growing Δ*asd* mutant strains. The following antibiotics were used as needed at the indicated concentrations: ampicillin, 100 μg/ml; chloramphenicol, 15 μg/ml; gentamicin, 20 μg/ml; kanamycin, 50 μg/ml; tetracycline, 10 μg/ml. Carbohydrate-free nutrient broth (NB) was used for growth when determining lipopolysaccharide (LPS) profiles. LB agar without sodium chloride and with 7.5% sucrose was employed for *sacB*-based counterselection. MacConkey agar plates with 1% mannose were used to indicate sugar fermentation.

For animal experiments, *S*. Typhimurium strains were grown in LB broth with appropriate supplements. Overnight cultures were diluted 1:100 and grown with shaking (200 rpm) to an optical density at 600 nm of ~0.8. Then, bacteria were centrifuged at 5,000 × *g* for 15 min at room temperature and resuspended in phosphate-buffered saline (PBS) or buffered saline with 0.01% gelatin (BSG) (40). LB or *Salmonella Shigella* (SS) agar plates were used to enumerate *S*. Typhimurium recovered from tissues. Selenite cystine broth was employed to enrich samples for *S*. Typhimurium. *Streptococcus pneumoniae* WU2 was cultured on brain heart infusion agar containing 5% sheep blood or in Todd-Hewitt broth with 0.5% yeast extract (41). All media, antibiotics, and chemicals were purchased from BD Difco (Franklin Lakes, NJ) or Sigma-Aldrich (St. Louis, MO).

### General DNA procedures.

DNA manipulations, including plasmid and genomic DNA isolation, restriction enzyme digestions, ligations, and other DNA-modifying reactions, were conducted as described previously (42) or were performed according to the manufacturers’ instructions (New England Biolabs, Ipswich, MA; Qiagen, Valencia, CA; Promega, Madison, WI). Synthesis of primers (Table S2) and DNA sequencing were performed by Integrated DNA Technologies (Coralville, IA) and the DNA Laboratory at Arizona State University (Tempe, AZ), respectively. PCRs were conducted with Klentaq LA polymerase (DNA Polymerase Technology, St. Louis, MO), possessing proofreading activity. Recombinant plasmids were introduced into *E. coli* and *S*. Typhimurium cells by transformation and electroporation, respectively.

### Construction of transcriptional *aph*-*lacZ* fusions.

DNA fragments containing the promoter regions of 12 fimbrial operons were amplified from the *S*. Typhimurium χ3761 genome by PCR using the appropriate primers (Table S2). The PCR products were digested with ApaI and BamHI and cloned into the unique ApaI/BamHI sites of *aph*-*lacZ* fusion suicide vector pSG3 (43). The resulting plasmids were introduced by conjugation into *S*. Typhimurium strain χ3761 to obtain fusions of selected promoter regions with *aph*-*lacZ* genes by a single-crossover event as previously described (43).

### *In vivo* expression technology (IVET).

Each *S*. Typhimurium *aph-lacZ* fusion strain was grown statically in LB broth at 37°C for 20 h. Bacterial cells were harvested by centrifugation at 5,000 × *g* for 15 min at room temperature. The pellets were resuspended in BSG buffer. BALB/c mice were inoculated orally with ~1 × 10^9^ CFU of the mixture of the 12 *aph-lacZ* fusion strains. Mice were treated with kanamycin at 3, 24, and 48 h postinoculation by oral administration (2 mg in 20 µl) and intraperitoneal (10 mg in 100 µl) injection. Three days after inoculation, the spleens were collected from the treated mice and homogenized. Dilutions of the homogenate were made in BSG and plated onto LB agar plates supplemented with gentamicin and incubated overnight at 37°C. Finally, selected clones were identified by PCR using specific primers (Table S2).

### Construction of suicide plasmids for introduction of deletions or ΔP_fimbrial operon_::P_*murA*_ deletion/insertions of fimbrial operons.

To construct the Δ*stiABCH1225*, Δ*safABCD31*, and Δ*stcABCD36* deletions, two-step PCR mutagenesis was used. First, two DNA fragments flanking fimbrial operons were amplified from the *S*. Typhimurium χ3761 genome using appropriate primer sets: PstiF/PstiR (P stands for primer, F stands for forward, and R stands for reverse) and d-stiAH-F/d-stiAH-R (d stands for deletion) (for Δ*stiABCH1225*), PsafF/PsafR and d-safAD-F/d-safAD-R (for Δ*safABCD31*), and PstcF/PstcR and d-stcAD-F/d-stiAD-R (for Δ*stiABCH1225*) (Table S2). Thereafter, the mixes of two PCR products flanking each fimbrial operon were used as the templates in the next amplification reactions with PstiF/d-stiAH-R, PsafF/d-safAD-R, and PstcF/d-stcAD-R primers, respectively. The DNA fragments obtained were digested with ApaI/SacI restriction enzymes and cloned into suicide plasmid vector pCHSUI-1. The resulting plasmids, pYA4584, pYA4586, and pYA5007, carried deletions of the entire *stiABCH*, *safABCD*, and *stcABCD* operons, respectively. Plasmids pYA3490 and pYA4941 for introduction of the *agfD812* and Δ(*agfC-agfG*)-*999* mutations were described previously (24, 44).

To construct the ΔP_*stiA52*_::P_*murA*_ *stiA52*, ΔP_*stcA53*_::P_*murA*_ *stcA53*, and ΔP_*safA55*_::P_*murA*_ *safA55* deletion/insertion mutations, two-step PCR mutagenesis was also used. DNA fragments containing the upstream regions of the *stiA*, *stcA*, and *safA* promoters were amplified from the *S*. Typhimurium χ3761 genome using PmurA-stiA-F/PmurA-stiA-R, PmurA-stcA-F/PmurA-stcA-R, and PmurA-safA-F/PmurA-safA-R primer pairs (Table S2), respectively. The PCR products were digested with BglII. A 65-bp *murA* promoter region was amplified from *E. coli* K-12 using primers Ec_PmurA-F and Ec_PmurA-R (Ec stands for *E*. *coli*). This PCR product was digested with BglII and NcoI. DNA fragments containing downstream regions of the *stiA*, *stcA*, and *safA* promoters were amplified from the *S*. Typhimurium χ3761 genome using PmurA-stiA-F1/PmurA-stiA-R1, PmurA-stcA-F1/PmurA-stcA-R1, and PmurA-safA-F1/PmurA-safA-R1 primer pairs, respectively. The PCR products were digested with NcoI. Two digested PCR products containing flanking regions of each fimbrial operon and a PCR product containing the *murA* promoter were combined by ligation and used as the templates for PCR to amplify the combined DNA fragments using PmurA-stiA-F/PmurA-stiA-R, PmurA-stcA-F/PmurA-stcA-R1, and PmurA-safA-F/PmurA-safA-R1 primer pairs, respectively. These final PCR products were digested with KpnI and SacI and cloned into the unique KpnI/SacI sites of suicide vector pRE112 to generate pYA5052, pYA5053, and pYA5054.

### Construction of *S*. Typhimurium mutants.

All *S*. Typhimurium mutants were derived from the highly virulent parent strain χ3761 (45). The genealogy of constructed strains is shown in Table S1. All gene replacements were introduced by conjugational transfer of suicide plasmids using donor *E. coli* strain χ7213 (46). All mutations were verified by PCR. We confirmed arabinose-regulated Fur production by Western blotting. The Δ*pmi* mutation was confirmed by white colony phenotype on mannose-MacConkey agar. Lipopolysaccharide (LPS) profiles were examined by silver staining of 12% polyacrylamide gels as described previously (47).

10.1128/mBio.01189-17.2 TABLE S1  Additional strains and plasmids used in this study. Download TABLE S1, DOCX file, 0.03 MB. Copyright © 2017 Łaniewski et al. 2017 Łaniewski et al. This content is distributed under the terms of the Creative Commons Attribution 4.0 International license.

10.1128/mBio.01189-17.3 TABLE S2  Primers used in this study. Download TABLE S2, DOCX file, 0.02 MB. Copyright © 2017 Łaniewski et al. 2017 Łaniewski et al. This content is distributed under the terms of the Creative Commons Attribution 4.0 International license.

### SDS-PAGE and Western blotting.

SDS-PAGE and Western blotting were performed by standard techniques. The blots were developed with nitroblue tetrazolium chloride/5-bromo-4-chloro-3′-indolyl phosphate (Amresco, Solon, OH) or Pierce ECL Western blotting substrate (Thermo Scientific), using rabbit polyclonal anti-rPspA serum as primary antibodies and mouse anti-rabbit IgG alkaline phosphatase conjugate (Sigma-Aldrich) as secondary antibodies.

### Animal supply and housing.

Female BALB/c mice (6 to 8 weeks old) were obtained from Charles River Laboratories (Wilmington, MA). Animals were allowed to acclimate for 1 week after arrival before starting the experiments. All animal procedures were carried out in compliance with the Institutional Animal Care and Use Committee (IACUC) at Arizona State University and the Animal Welfare Act.

### Colonization of the mouse spleen and determination of the competitive index.

BALB/c mice were inoculated intraperitoneally with a mixture containing ~1 × 10^4^ of S. Typhimurium wild-type strain (χ3761) and either strain χ11484 or strain χ11599 suspended in 100 μl of PBS. Wild-type and mutant strains were marked with low-copy-number chloramphenicol- or kanamycin-resistant plasmids: pHSG576 and pWSK129, respectively. On days 1 and 3 postinoculation, three mice in each group were euthanized, and the spleens and livers were collected to determine the colonization levels. The competitive index (CI) for each strain compared to the wild type was calculated by dividing the ratio of two strains from an organ divided by the same ratio in the suspension used for the infection.

### Determination of the 50% lethal dose.

Freshly grown bacterial cultures were pelleted by centrifugation at 5,000 × *g* for 15 min at room temperature. Bacterial pellets were resuspended in BSG and adjusted to achieve a dose of ~10^2^ to ~10^9^ CFU in a volume of 20 μl for orally inoculating BALB/c mice. Animals were observed for typhoid symptoms for 3 weeks postinoculation. Deaths were recorded daily. The 50% lethal dose (LD_50_) was calculated using the Reed and Muench method (48).

### Immunization and pneumococcal challenge.

BALB/c mice were inoculated orally with 20 μl of PBS containing ~1 × 10^8^ CFU of the appropriate *S*. Typhimurium strain and boosted with the same strain and dose 6 weeks later. No food or water was provided for ~4 h prior to immunizations. Groups of mice inoculated with PBS served as a control. At week 10 (i.e., 4 weeks after the booster), all mice were challenged by intraperitoneal injection with ~1 × 10^4^ CFU of *S. pneumoniae* WU2 in 100 µl of BSG (equivalent to 40 times the LD_50_). Mice were monitored daily for 3 weeks.

### Antigen preparation and ELISA.

Recombinant PspA (rPspA) protein was purified from *E. coli* BL21(DE3)(pYA4085) as described previously (49). Antibody titers in serum and vaginal washes were determined by enzyme-linked immunosorbent assay (ELISA) as described previously (50).

### Statistical analyses.

All statistical analyses were performed using GraphPad Prism 6 (GraphPad Software, San Diego, CA). The significance of the different values obtained was appraised using two-way analysis of variance (ANOVA) followed by Dunnett’s tests (for ELISA). For challenge experiments, log rank (Mantel-Cox) test was used to determine the significant differences between the survival curves. For CI assays, the geometric means of the CIs were determined, and a Student’s *t* test was used to determine whether the logarithmically transformed ratios differed significantly from zero. *P* values of <0.05 were considered significant.
