# Supplementary figures and images for: Analysis of Spleen-Induced Fimbria Production in Recombinant Attenuated Salmonella enterica Serovar Typhimurium Vaccine Strains
Source: mBio. 2017 Aug 22;8(4):e01189-17. doi: 10.1128/mBio.01189-17 (PMC5565968; doi:10.1128/mBio.01189-17)

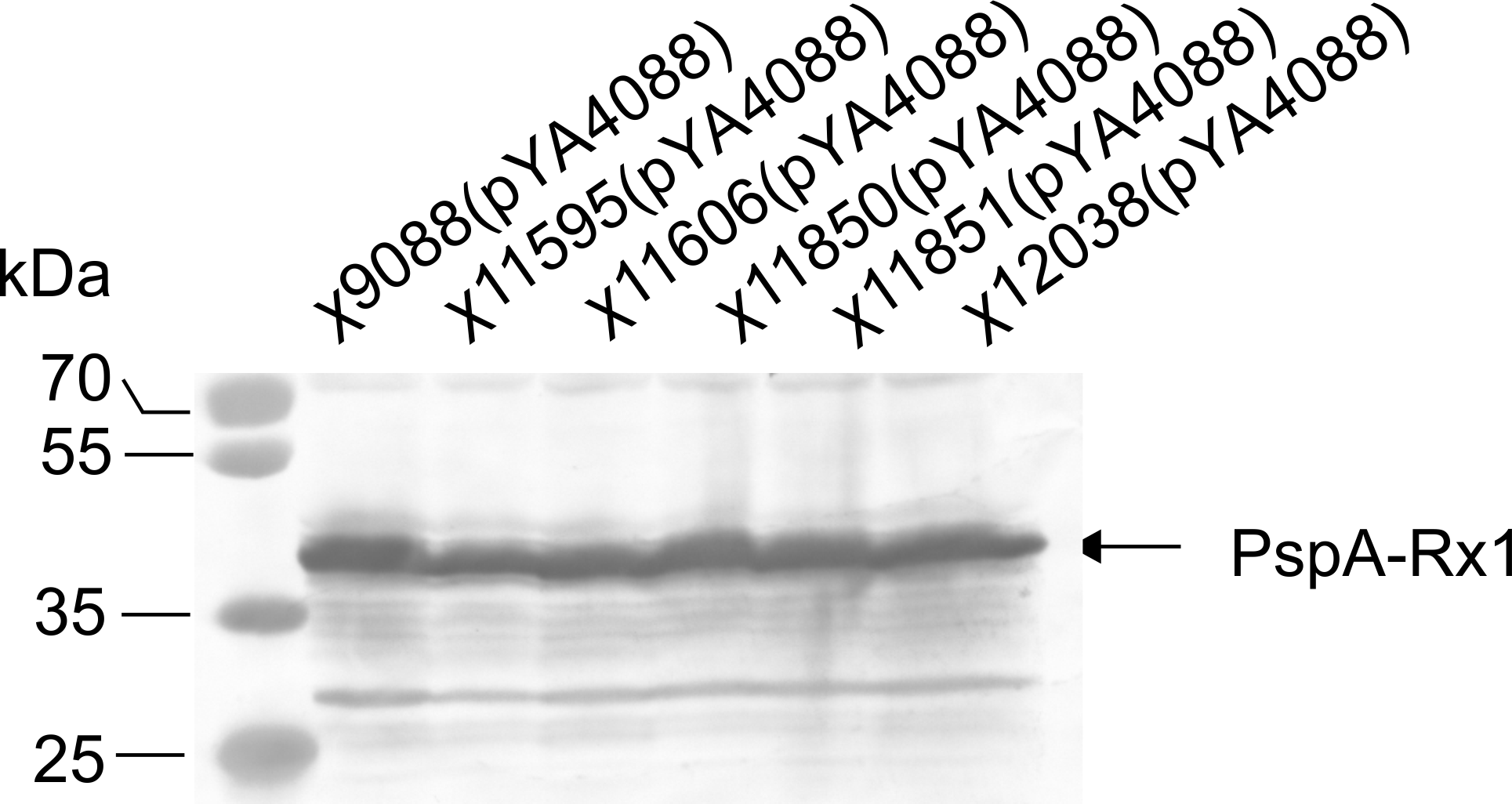

Supplement: FIG S1 [file mbo004173452sf1.tif]
