# Supplementary material for: Analysis of Spleen-Induced Fimbria Production in Recombinant Attenuated Salmonella enterica Serovar Typhimurium Vaccine Strains
Source: mBio. 2017 Aug 22;8(4):e01189-17. doi: 10.1128/mBio.01189-17 (PMC5565968; doi:10.1128/mBio.01189-17)
Supplement: TABLE S1 [file mbo004173452st1.docx]

**Table S1.** Additional strains and plasmids.

| **Name** | **Relevant characteristics** | **Source or parent strain** |
| --- | --- | --- |
| *S.* Typhimurium strains |  |  |
| χ9450 | P_bcf_::pYA4306 | χ3761 |
| χ9451 | P_sti_::pYA4307 | χ3761 |
| χ9452 | P_stf_::pYA4308 | χ3761 |
| χ9453 | P_saf_::pYA4309 | χ3761 |
| χ9454 | P_stb_::pYA4310 | χ3761 |
| χ9455 | P_fim_::pYA4311 | χ3761 |
| χ9456 | P_agf_::pYA4312 | χ3761 |
| χ9457 | P_std_::pYA4314 | χ3761 |
| χ9458 | P_lpf_::pYA4315 | χ3761 |
| χ9459 | P_sth_::pYA4316 | χ3761 |
| χ9460 | P_pef_::pYA4317 | χ3761 |
| χ9461 | P_stc_::pYA4313 | χ3761 |
| χ11351 | Δ(*agfC-agfG*)*-999* | χ3761; Soo-Young Wanda |
| χ11430 | Δ*stiABCH1225* | χ3761 |
| χ11432 | Δ*safABCD31* | χ3761 |
| χ11438 | Δ*stcABCD36* | χ3761 |
| χ11466 | Δ(*agfC-agfG*)*-999* Δ*safABCD31* | χ11351 |
| χ11468 | Δ*safABCD31* Δ*stiABCH1225* | χ11432 |
| χ11506 | Δ(*agfC-agfG*)*-999* Δ*stiABCH1225* | χ11351 |
| χ11559 | Δ*pmi-2426* Δ(*gmd-fcl*)*-26* ΔP_fur33_::TT *araC* P_BAD_ *fur* Δ*asdA33* Δ*stiABCH1225* | χ9088 |
| χ11560 | Δ*pmi-2426* Δ(*gmd-fcl*)*-26* ΔP_fur33_::TT *araC* P_BAD_ *fur* Δ*asdA33* Δ*safABCD31* | χ9088 |
| χ11561 | Δ*pmi-2426* Δ(*gmd-fcl*)*-26* ΔP_fur33_::TT *araC* P_BAD_ *fur* Δ*asdA33* Δ*safABCD31* Δ*stiABCH1225* | χ11560 |
| χ11562 | Δ*pmi-2426* Δ(*gmd-fcl*)*-26* ΔP_fur33_::TT *araC* P_BAD_ *fur* Δ*asdA33* Δ*safABCD31* Δ*stiABCH1225* Δ*stcABCD36* | χ11561 |
| χ11590 | Δ*pmi-2426* Δ(*gmd-fcl*)*-26* ΔP_fur33_::TT *araC* P_BAD_ *fur* Δ*asdA33* Δ*stiABCH1225* Δ(*agfC-agfG*)*-999* | χ11559 |
| χ11591 | Δ*pmi-2426* Δ(*gmd-fcl*)*-26* ΔP_fur33_::TT *araC* P_BAD_ *fur* Δ*asdA33* Δ*safABCD31* Δ(*agfC-agfG*)*-999* | χ11560 |
| χ11592 | Δ*pmi-2426* Δ(*gmd-fcl*)*-26* ΔP_fur33_::TT *araC* P_BAD_ *fur* Δ*asdA33* Δ*stiABCH1225* Δ(*agfC-agfG*)*-999* Δ*stcABCD36* | χ11590 |
| χ11594 | Δ*pmi-2426* Δ(*gmd-fcl*)*-26* ΔP_fur33_::TT *araC* P_BAD_ *fur* Δ*asdA33* Δ*safABCD31* Δ(*agfC-agfG*)*-999* Δ*stcABCD36* | χ11591 |
| χ11597 | Δ*pmi-2426* Δ(*gmd-fcl*)*-26* ΔP_fur33_::TT *araC* P_BAD_ *fur* Δ*asdA33* Δ*safABCD31* Δ*stiABCH1225* Δ(*agfC-agfG*)*-999* | χ11561 |
|  |  |  |
| *Plasmids used for IVET* | | |
| pYA4306 | pSG3 derivative for introduction P_bcf_::*aph lacZ* | This study |
| pYA4307 | pSG3 derivative for introduction P_sti_::*aph lacZ* | This study |
| pYA4308 | pSG3 derivative for introduction P_stf_::*aph lacZ* | This study |
| pYA4309 | pSG3 derivative for introduction P_saf_::*aph lacZ* | This study |
| pYA4310 | pSG3 derivative for introduction P_stb_::*aph lacZ* | This study |
| pYA4311 | pSG3 derivative for introduction P_fim_::*aph lacZ* | This study |
| pYA4312 | pSG3 derivative for introduction P_agf_::*aph lacZ* | This study |
| pYA4313 | pSG3 derivative for introduction P_stc_::*aph lacZ* | This study |
| pYA4314 | pSG3 derivative for introduction P_std_::*aph lacZ* | This study |
| pYA4315 | pSG3 derivative for introduction P_lpf_::*aph lacZ* | This study |
| pYA4316 | pSG3 derivative for introduction P_sth_::*aph lacZ* | This study |
| pYA4317 | pSG3 derivative for introduction P_pef_::*aph lacZ* | This study |
|  |  |  |
| *Plasmids used for introduction of mutations* | |  |
| pRE112 | suicide vector; *sacB* *mobRP4*; R6K *ori*; Cm^R^ | (1) |
| pDMS197 | suicide vector; *sacB* *mobRP4*; R6K *ori*; Tc^R^ | (1) |
| pCHSUI-1 | suicide vector; *lacZα sacR sacB* *mobRP4*; R6K *ori*; Tc^R^ | (2) |
| pYA3490 | pDMS197 derivative for introduction *agfD812* | (3) |
| pYA4941 | pRE112 derivative for introduction Δ(*agfC-agfG*)*-999* | (4) |
| pYA5007 | pCHSUI-1 derivative for introduction Δ*stcABCD36* | This study |
| pYA5052 | pRE112 derivative for introduction ΔP_stiA52_::P_murA_ *stiA52* | This study |
| pYA4584 | pCHSUI-1 derivative for introduction Δ*stiABCH1225* | This study |
| pYA4586 | pCHSUI-1 Δ*safABCD31* | This study |
| pYA5007 | pCHSUI-1 Δ*stcABCD36* | This study |
| pYA5052 | pRE112 ΔP_stiA52_::P_murA_ *stiA52* | This study |
| pYA5053 | pRE112 ΔP_stcA53_::P_murA_ *stcA53* | This study |
| pYA5055 | pRE112 ΔP_safA55_::P_murA_ *safA55* | This study |
|  |  |  |
| *Plasmids used for coinfection studies* | |  |
| pHSG576 | pHSG576 | (5) |
| pWSK129 | pWSK129 | (6) |

**References**

1. **Edwards, R. A., L. H. Keller, and D. M. Schifferli.** 1998. Improved allelic exchange vectors and their use to analyze 987P fimbria gene expression. Gene **207:**149-157.

2. **Baek, C. H., S. Wang, K. L. Roland, and R. Curtiss, 3rd.** 2009. Leucine-responsive regulatory protein (Lrp) acts as a virulence repressor in *Salmonella enterica* serovar Typhimurium. J Bacteriol **191:**1278-1292.

3. **Kang, H. Y., C. M. Dozois, S. A. Tinge, T. H. Lee, and R. Curtiss, 3rd.** 2002. Transduction-mediated transfer of unmarked deletion and point mutations through use of counterselectable suicide vectors. J Bacteriol **184:**307-312.

4. **Sun, W., J. Olinzock, S. Wang, S. Sanapala, and R. Curtiss, 3rd.** 2014. Evaluation of YadC protein delivered by live attenuated *Salmonella* as a vaccine against plague. Pathog Dis **70:**119-131.

5. **Takeshita, S., M. Sato, M. Toba, W. Masahashi, and T. Hashimoto-Gotoh.** 1987. High-copy-number and low-copy-number plasmid vectors for *lacZ* lalpha-complementation and chloramphenicol- or kanamycin-resistance selection. Gene **61:**63-74.

6. **Wang, R. F., and S. R. Kushner.** 1991. Construction of versatile low-copy-number vectors for cloning, sequencing and gene expression in *Escherichia coli*. Gene **100:**195-199.
