# Supplementary material for: Analysis of Spleen-Induced Fimbria Production in Recombinant Attenuated Salmonella enterica Serovar Typhimurium Vaccine Strains
Source: mBio. 2017 Aug 22;8(4):e01189-17. doi: 10.1128/mBio.01189-17 (PMC5565968; doi:10.1128/mBio.01189-17)
Supplement: TABLE S2 [file mbo004173452st2.docx]

**Table S2.** Primers used in this study.

| **Primer** | **Sequence (5’ to 3’)** | **Orientation** | **Restriction site** |
| --- | --- | --- | --- |
| *Primers used for construction* aph lacZ *fusions and deletions* | | | |
| PbcfF | ATGGGGCCC**TGAAGGTTGTTATGA** | Forward | ApaI |
| PbcfR | ACAGGATCC**TTCATTCAAGCTCCTT** | Reverse | BamHI |
| PstiF1 | TTTGGGCCC**GACAACACACTATG** | Forward | ApaI |
| PstiR | TAAGGATCC**TTTCATTATCTTTGATCC** | Reverse | BamHI |
| PstfF | TACGGGCCC**TTGCTATATATTCAGTAC** | Forward | ApaI |
| PstfR | GCTGGATCC**ATTTTTCATCCTTAT** | Reverse | BamHI |
| PsafF | TATGGGCCC**TTCGAAGATAAGTTTCC** | Forward | ApaI |
| PsafR | TGAGGATCC**ACCTTTTTTAAACC** | Reverse | BamHI |
| PstbF | ATCGGGCCC**TTTAACTGATACGGAT** | Forward | ApaI |
| PstbR | TTCGGATCC**ATATGATAATGTTCC** | Reverse | BamHI |
| PfimF | CGTGGGCCC**TCGTCGTTAATAG** | Forward | ApaI |
| PfimR | TTAGGATCC**ATGGATTTCCCTTGA** | Reverse | BamHI |
| PcsgBF | TTTGGGCCC**TGGCATTAACCTGGAC** | Forward | ApaI |
| PcsgBR | TTGGGATCC**ATGCTGTCACCCTGG** | Reverse | BamHI |
| PstdF | TTCGGGCCC**CTTATGATGGTTCATA** | Forward | ApaI |
| PstdR | AAAGGATCC**ACCTGAACTTTCCATC** | Reverse | BamHI |
| PlpfF | AGCGGGCCC**GTAACACATGCAACAC** | Forward | ApaI |
| PlpfR | AAAGGATCC**ATTTATGACGGTAG** | Reverse | BamHI |
| PsthF | AACGGGCCC**GTTTCATTTTAAACGG** | Forward | ApaI |
| PsthR | TTAGGATCC**ATATTAATTGTTGCC** | Reverse | BamHI |
| PpefF | CATGGGCCC**GTGTTGTTCACGGACT** | Forward | ApaI |
| PpefR | CTCGGATCC**ATGTATATAACCTCT** | Reverse | BamHI |
| PstcF | ATAGGGCCC**GACAGATTGTTGT** | Forward | ApaI |
| PstcR | GTTGGATCC**TCTCTTCCTTAATGAAG** | Reverse | BamHI |
| d-stiAH-F | TTCGGATCC**GCTGAGAGAAGCGTT** | Forward | BamHI |
| d-stiAH-R | CTCGAGCTC**GTGCCTTTCCAGGTGC** | Reverse | SacI |
| d-safAD-F | TACGGATCC**GTCTGTTCCACTCATAC** | Forward | BamHI |
| d-safAD-R | TTTGAGCTC**AGATTCGGCCAGCCATTTC** | Reverse | SacI |
| d-stcAD-F | CTAGGATCC**ACTGCGCTGATCTATGTG** | Forward | BamHI |
| d-stcAD-R | CCGGAGCTC**AAATGTACCGCACCGATG** | Reverse | SacI |
| *Primers used for construction deletion/insertions* | | | |
| Ec_PmurA-F | TTGAGATCT**GGGCGATTCGCCGGTAGCGGATATGAATTG** | Forward | BglII |
| Ec_PmurA-R | TATCCATGG**AGTTTGTTCTCAGTTAACAATTCATATCC** | Reverse | NcoI |
| PmurA-stiA-F | CCGGAGCTC**TCGGTTAAAAATATCACGCCTTC** | Forward | SacI |
| PmurA-stiA-R | TAAAGA TCT**CGCGAAGAAAAATTCAGAATAC** | Reverse | BglII |
| PmurA-stiA-F1 | AGACCATGG**AACTCTCCTTAAAAACACTCACTG** | Forward | NcoI |
| PmurA-stiA-R1 | ACTGGTACC**GACCATGCCTGAAGATCG** | Reverse | KpnI |
| PmurA-stcA-F | ATAGAGCTC**GCATTGTTAATTCCTGCT** | Forward | SacI |
| PmurA-stcA-R | TAAAGATCT**TTAGTCGTAATGATTAACAAC** | Reverse | BglII |
| PmurA-stcA-F1 | AGACCATGG**AACGTTCACTTATTGCTG** | Forward | NcoI |
| PmurA-stcA-R1 | TGAGGTACC**ATCACCAGAAGCAACATC** | Reverse | KpnI |
| PmurA-safA-F | CAAGAG CTC**CCATTAAAGATCTTCCCTGC** | Forward | SacI |
| PmurA-safA-R | AACAGATCT**ATGACACCTACTACACTG** | Reverse | BglII |
| PmurA- safA-F1 | AAACCATGG**TTATTCAAATGAAAAGCAC** | Forward | NcoI |
| PmurA- safA-R1 | ATAGGTACC**GTGGCGAACAAAGCTACC** | Reverse | KpnI |
